# Changes in clinical and imaging variables during withdrawal of heart failure therapy in recovered dilated cardiomyopathy

**DOI:** 10.1002/ehf2.13872

**Published:** 2022-03-08

**Authors:** Brian P. Halliday, Ruth Owen, John Gregson, Ali Vazir, Rebecca Wassall, Zohya Khalique, Amrit S. Lota, Upasana Tayal, Daniel J. Hammersley, Richard E. Jones, Dudley J. Pennell, Martin R. Cowie, John G.F. Cleland, Sanjay K. Prasad

**Affiliations:** ^1^ Cardiovascular Research Centre, Royal Brompton Hospital, Guy's and St Thomas' NHS Trust and National Heart Lung Institute Imperial College London SW3 6NP UK; ^2^ Department of Medical Statistics London School of Hygiene and Tropical Medicine London UK; ^3^ Faculty of Life Sciences & Medicine King's College London London UK; ^4^ Robertson Centre for Biostatistics University of Glasgow Glasgow UK

**Keywords:** Dilated cardiomyopathy, Myocardial recovery, Remission, Remodelling

## Abstract

**Aims:**

This study aimed to profile the changes in non‐invasive clinical, biochemical, and imaging markers during withdrawal of therapy in patients with recovered dilated cardiomyopathy, providing insights into the pathophysiology of relapse.

**Methods and results:**

Clinical, biochemical, and imaging data from patients during phased withdrawal of therapy in the randomized or single‐arm cross‐over phases of TRED‐HF were profiled. Clinical variables were measured at each study visit and imaging variables were measured at baseline, 16 weeks, and 6 months. Amongst the 49 patients [35% women, mean age 53.6 years (standard deviation 11.6)] who withdrew therapy, 20 relapsed. Increases in mean heart rate [7.6 beats per minute (95% confidence interval, CI, 4.5, 10.7)], systolic blood pressure [6.6 mmHg (95% CI 2.7, 10.5)], and diastolic blood pressure [5.8 mmHg (95% CI 3.1, 8.5)] were observed within 4–8 weeks of starting to withdraw therapy. A rise in mean left ventricular (LV) mass [5.1 g/m^2^ (95% CI 2.8, 7.3)] and LV end‐diastolic volume [3.9 mL/m^2^ (95% CI 1.1, 6.7)] and a reduction in mean LV ejection fraction [−4.2 (95% CI −6.6, −1.8)] were seen by 16 weeks, the earliest imaging follow‐up. Plasma N‐terminal pro‐brain natriuretic peptide (NT‐proBNP) fell immediately after withdrawing beta‐blockers and only tended to increase 6 months after beginning therapy withdrawal [mean change in log NT‐proBNP at 6 months: 0.2 (95% CI −0.1, 0.4)].

**Conclusions:**

Changes in plasma NT‐proBNP are a late feature of relapse, often months after a reduction in LV function. A rise in heart rate and blood pressure is observed soon after withdrawing therapy in recovered dilated cardiomyopathy, typically accompanied or closely followed by early changes in LV structure and function.

## Introduction

Left ventricular (LV) reverse remodelling frequently occurs amongst patients with dilated cardiomyopathy (DCM).^1^, ^2^ With increasing therapeutic success, the number of patients with heart failure and improved left ventricular ejection fraction (LVEF) is growing. Although improvement in LVEF is associated with a much better prognosis, these patients remain at risk of adverse outcomes, including a relapse of cardiac dysfunction and symptoms of heart failure.^3^, ^4^, ^5^ This is more likely to occur if medication is withdrawn but also in a minority who continue on therapy.^3^, ^4^, ^5^ Priorities for future research include identifying markers of true and sustained recovery as well as features of early relapse. This may provide insights into the drivers of relapse allowing therapy to be tailored appropriately and identifying those most at risk. Understanding the sequence of changes that occur during adverse remodelling and relapse is an important step towards these goals.

A previous report from the TRED‐HF trial suggested that a greater rise in heart rate during therapy withdrawal was associated with a higher risk of relapse.^6^ Changes in other clinical, imaging, or biochemical markers during therapy withdrawal will provide useful insights into the pathophysiology of remodelling as well as possible indicators of relapse that may be used in clinical practice; whether such variables are effective monitoring options is unknown. Whether changes in natriuretic peptides precede or parallel structural remodelling in this population will inform the use of this marker in future studies and clinical practice.

In this study, we profiled the serial changes in non‐invasive clinical, biochemical, and imaging markers during withdrawal of therapy in patients with recovered DCM to provide insights into the sequence of changes that occur during adverse remodelling and relapse.

## Methods

The TRED‐HF was an open‐label, randomized trial examining the safety and feasibility of phased withdrawal of pharmacological therapy for heart failure in asymptomatic patients with DCM and improved LVEF and low plasma concentrations of natriuretic peptides (NCT02859311).^3^, ^6^ All patients provided informed consent. The study was approved by the London‐Surrey Borders National Research Ethics Committee and authorized by the Medicine and Healthcare Products Regulatory Agency.

Overall, 51 patients with a previous diagnosis of DCM, whose LVEF had improved from <40% to ≥50% and who now had normal left ventricular end‐diastolic volume (LVEDV) and plasma N‐terminal pro‐brain natriuretic peptide (NT‐proBNP) < 250 ng/L and who were still taking at least one heart failure therapy [loop diuretic, beta‐blocker, angiotensin‐converting enzyme (ACE) inhibitor, angiotensin receptor blocker (ARB), or mineralocorticoid receptor antagonist (MRA)] were included. Patients were randomized 1:1 to phased withdrawal or continuation of heart failure therapy for 6 months. After 6 months, patients assigned to the control arm subsequently entered a single‐arm cross‐over phase. Between Months 6 and 12, they had therapy withdrawn in the same fashion as the randomized phase of the study.

Therapy withdrawal was done in a step‐wise fashion over a maximum of 16 weeks. Changes were made every 2 weeks following review by the study team. Heart rate, blood pressure, and plasma concentration of NT‐proBNP were measured every 4 weeks with interim reviews taking place via telephone, if feasible and safe. All patients had cardiovascular magnetic resonance (CMR) at baseline, 16 weeks, and 6 months with gold‐standard volumetric assessment of cardiac chambers by two EACVI Level 3 accredited operators.^3^, ^7^ Serial scans from the same participant were analysed by the same operator. Two patients with devices had imaging follow‐up with three‐dimensional echocardiography. Serial LVEF and left ventricular end‐diastolic volume indexed to body surface area (LVEDVi) are included. Two patients who initially had CMR were unable to continue follow‐up with this modality due to new contraindications. Follow‐up LVEF is included from three‐dimensional echocardiography for these patients. No other volumetric measures are included given the poor between modality reproducibility.^8^, ^9^

Loop diuretics were withdrawn first, followed by MRAs, beta‐blockers, and finally ACE inhibitors or ARBs at separate visits. The medication was stopped if the participant was taking 40 mg or less of furosemide (or equivalent), 50 mg or less of spironolactone, or 25% or less of the recommended dose of beta‐blocker or ACE inhibitor or ARB. If participants were prescribed larger doses of any of the above, these were reduced by 50%, rather than stopped, every 2 weeks.

The primary relapse endpoint was defined by any one of the following: (i) a reduction in LVEF by >10% and to <50%, or (ii) an increase in LVEDV by >10% and to above the normal range, or (iii) a two‐fold rise in NT‐proBNP from baseline and to >400 ng/L, or (iv) clinical evidence of heart failure. Treatment was restarted if any of the primary endpoint criteria were met. The management of patients who suffered adverse events but did not meet the primary endpoint was determined by the research team and the patient's physicians.

### Statistical analysis

The mean and mean change from baseline are presented at the different time points for each variable with 95% confidence intervals (CIs). Patient data were included until the end of the study, which was defined as the final follow‐up visit or the time at which the primary endpoint was first met. Change from baseline was analysed using paired *t*‐tests. Percentage change in variables from baseline along with 95% CIs are presented at different time points. The same data are presented stratified by the occurrence of relapse in the supplementary data for those variables not included in the primary endpoint composite. Serial changes in clinical variables collected at each study visit were modelled using fractional polynomial models with four knots. We also examined changes in NT‐proBNP before, during, and after the withdrawal of beta‐blockers and ACE inhibitors/ARBs, individually.

Statistical analyses were performed using Stata Version 16.0 (StatCorp, College Station, TX, USA). The investigators had complete access to all raw and derived datasets.

## Results

Of the 51 patients enrolled and randomized, 49 attempted withdrawal of therapy during the study; 25 patients were randomized to have therapy withdrawn between 0 and 6 months, whilst 24 of 26 patients initially randomized to continue therapy, attempted to withdraw therapy in the single‐arm cross‐over phase between 6 and 12 months. Of the 49 patients who withdrew therapy, 20 (41%) met the primary endpoint for relapse. Of the 20 patients who relapsed, 10 fulfilled more than one element of the primary composite relapse endpoint; 12 (60%) met the LVEF criterion, 11 (55%) the LVEDVi criterion, 9 (45%) the NT‐proBNP criterion, and 1 (5%) developed peripheral oedema. Four participants restarted therapy without meeting the primary endpoint, two for hypertension, one following an episode of atrial fibrillation, and one following an episode of non‐sustained ventricular tachycardia. Baseline characteristics are presented in *Table* *1*. Around half of participants were neither on a loop diuretic nor MRA at baseline, and consequently, beta‐blockers were the first treatment to be withdrawn.

**Table 1:** Baseline characteristics

	All patients who had therapy withdrawn
	*N* = 49
**Demographics**	
Age (years)	53.6 (11.6)
Women	17 (35)
Heart rate (b.p.m.)	66.4 (11.0)
Systolic BP (mmHg)	124 (12)
Diastolic BP (mmHg)	72 (10)
Weight (kg)	86 (22)
**Co‐morbidities**	
Previous AF	11 (22)
Hypertension	4 (8)
Diabetes mellitus	1 (2)
**Aetiology**	
Idiopathic	34 (69)
Familial	6 (12)
Environmental insult	9 (18)
TTNtv	10 (20)
**Medications**	
ACE inhibitor/ARB	49 (100)
Beta‐blocker	43 (88)
MRA	23 (47)
Loop diuretic	6 (12)
**Imaging**	
LVEF (%)	60 (6)
LVEDVi (mL/m^2^)	79 (15)
RVEF (%)	58 (6)
RVEDVi (mL/m^2^)	78 (17)
LAVi (mL/m^2^)	40 (9)
LVMi (g/m^2^)	68 (15)
**Biomarkers and symptoms**	
NT‐proBNP (ng/L)	76 (40 127)

ACE, angiotensin‐converting enzyme; AF, atrial fibrillation; ARB, angiotensin receptor blocker; BP, blood pressure; LAVi, left atrial volume indexed to body surface area; LVEDVi, left ventricular end‐diastolic volume indexed to body surface area; LVEF, left ventricular ejection fraction; LVMi, left ventricular mass indexed to body surface area; MRA, mineralocorticoid receptor blocker; NT‐proBNP, N‐terminal pro‐brain natriuretic peptide; RVEDVi, right ventricular end‐diastolic volume indexed to body surface area; RVEF, right ventricular ejection fraction; TTNtv, truncating genetic variants in *TTN*.

Data are presented as mean (standard deviation), median (interquartile range), or *n* (%). Characteristics are taken at baseline for patients in the randomized phase and at 6 months for those having therapy withdrawn in cross‐over phase.

### Clinical variables

There was a rise in mean heart rate between baseline and 4 weeks [66.1 beats per minute (b.p.m.) (95% CI 63.0, 69.3) to 73.7 b.p.m. (95% CI 70.3, 77.2); change from baseline 7.6 b.p.m. (95% CI 4.5, 10.7); *P* < 0.0001], which was followed by a rise in systolic and diastolic blood pressure between baseline and 8 weeks [systolic 124.0 mmHg (95% CI 120.7, 127.3) to 130.8 mmHg (95% CI 126.9, 134.7), change from baseline 6.6 mmHg (95% CI 2.7, 10.5); *P* = 0.001; diastolic 72.3 mmHg (95% CI 69.5, 75.1) to 77.7 mmHg (95% CI 74.5, 80.9), change from baseline 5.8 mmHg (95% CI 3.1, 8.5); *P* < 0.001] (Supporting Information, *Figure S1* and *Table* *S1*).

**Figure 1:**
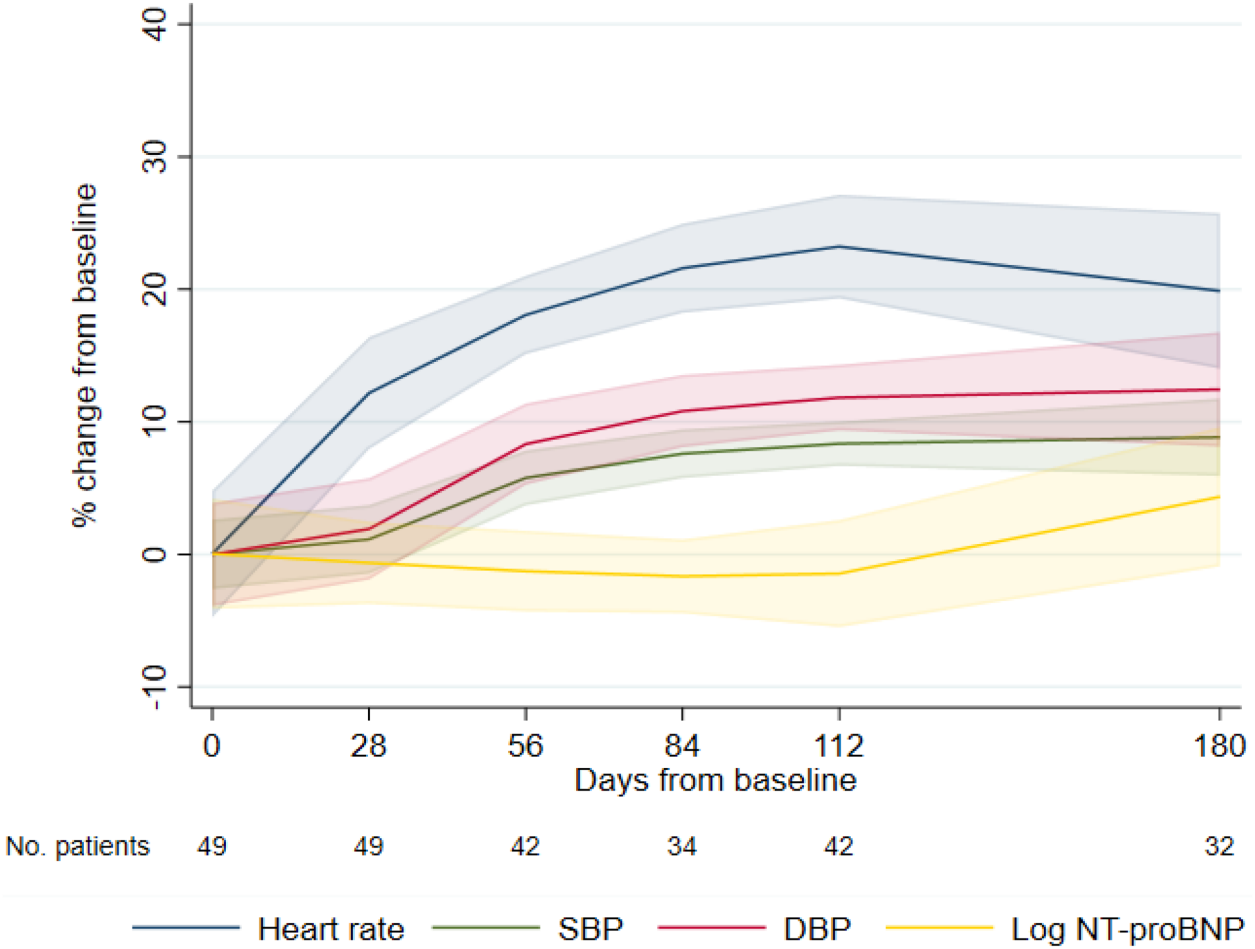
Mean percentage change from baseline in clinical and biochemical variables modelled using fractional polynomials until the end of the study or relapse. DBP, diastolic blood pressure; NT‐proBNP, N‐terminal pro‐brain natriuretic peptide; SBP, systolic blood pressure.

Mean log NT‐proBNP remained similar over 6 months [4.2 (95% CI 4.0, 4.4) to 4.2 (95% CI 3.8, 4.5)], with a non‐significant trend to increase from baseline seen at 6 months [0.2 (95% CI −0.1, 0.4); *P* = 0.18] (*Figure* *1*). As expected, the mean change from baseline in log NT‐proBNP was most pronounced in those who met the primary relapse endpoint [0.9 (95% CI 0.0, 1.8)] (Supporting Information, *Figure S1* and *Table* *S1*). However, of those who met the relapse endpoint, seven had a reduction in NT‐proBNP at the point of relapse compared with baseline, five of whom had a reduction in LVEF of >10%.

### Imaging variables

**Figure 2:**
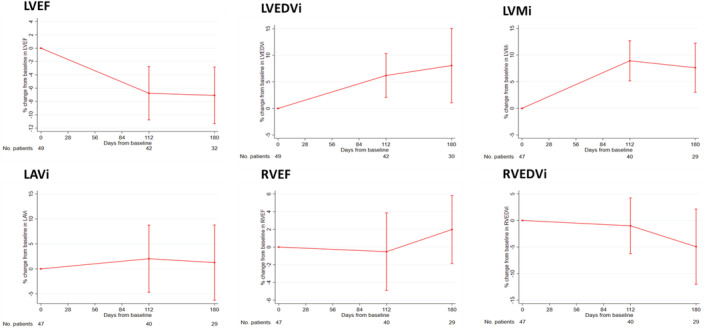
Mean percentage change from baseline in cardiovascular magnetic resonance variables until the end of the study or relapse. LAVi, left atrial volume indexed to body surface area; LVEDVi, left ventricular end‐diastolic volume indexed to body surface area; LVEF, left ventricular ejection fraction; LVMi, left ventricular mass indexed to body surface area; RVEDVi, right ventricular end‐diastolic volume indexed to body surface area; RVEF, right ventricular ejection fraction.

There was a reduction in LVEF between baseline and 16 weeks [59.9% (95% CI 58.2, 61.6) to 55.2% (95% CI 52.7, 57.6); change from baseline −4.2% (95% CI −6.6, −1.8), *P* < 0.001], which was accompanied by an increase in LVEDVi [79.0 mL/m^2^ (95% CI 74.7, 83.3) to 81.40 mL/m^2^ (95% CI 76.9, 85.9); change from baseline 3.9 mL/m^2^ (95% CI 1.1, 6.7), *P* = 0.008] and left ventricular mass indexed to body surface area (LVMi) [67.8 g/m^2^ (95% CI 63.2, 72.3) to 72.7 g/m^2^ (95% CI 67.9, 77.5); change from baseline 5.1 g/m^2^ (95% CI 2.8, 7.3), *P* < 0.001] (*Figure* *2* and *Table* *2*). Mean left atrial volume indexed to body surface area (LAVi) and right ventricular ejection fraction (RVEF) remained similar over 6 months, although there was an increase in mean LAVi amongst those who relapsed at 6 months (Supporting Information, *Table S1* and *Figure* *S1*). There was a reduction in right ventricular end‐diastolic volume indexed to body surface area (RVEDVi) that was greatest after 6 months [change from baseline −5.8 mL/m^2^ (95% CI −9.9, −1.6), *P* = 0.009] (*Figure* *2* and *Table* *2*).

**Table 2:** Clinical, biochemical, and imaging variables at baseline and follow‐up

		Heart rate		SBP		DBP		Log NT‐proBNP		LVEF	
	*n*	Mean (95% CI)	*P*	Mean (95% CI)	*P*	Mean (95% CI)	*P*	Mean (95% CI)	*P*	Mean (95% CI)	*P*
Baseline	49	66.1 (63.0, 69.3)		124.0 (120.7, 127.3)		72.3 (69.5, 75.1)		4.2 (4.0, 4.4)		59.9 (58.2, 61.6)	
28 days	49	73.7 (70.3, 77.2)		125.0 (121.0, 129.1)		72.9 (69.9, 75.8)		4.2 (3.9, 4.5)			
Change from baseline	49	7.6 (4.5, 10.7)	<0.001	1.0 (−2.5, 4.6)	0.55	0.6 (−2.3, 3.4)	0.69	−0.0 (−0.2, 0.2)	0.89		
% change from baseline	49	12.7 (7.7, 17.8)		1.1 (−1.9, 4.1)		1.7 (−2.5, 6.0)		−0.2 (−5.2, 4.8)			
56 days	42	76.9 (73.3, 80.6)		130.8 (126.9, 134.7)		77.7 (74.5, 80.9)		4.1 (3.8, 4.4)			
Change from baseline	42	9.5 (5.6, 13.5)	<0.001	6.6 (2.7, 10.5)	0.001	5.8 (3.1, 8.5)	<0.001	−0.1 (−0.3, 0.1)	0.27		
% change from baseline	42	15.9 (9.4, 22.3)		5.7 (2.5, 9.0)		8.8 (4.8, 12.7)		−2.6 (−7.3, 2.1)			
84 days	34	82.0 (77.4, 86.6)		133.9 (130.2, 137.7)		80.5 (77.2, 83.8)		4.3 (4.0, 4.6)			
Change from baseline	34	15.7 (11.4, 20.1)	<0.001	9.6 (5.8, 13.3)	<0.001	7.8 (4.3, 11.4)	<0.001	−0.0 (−0.3, 0.3)	1.00		
% change from baseline	34	25.3 (18.0, 32.5)		8.1 (5.0, 11.3)		11.9 (6.7, 17.1)		0.3 (−5.6, 6.2)			
112 days	42	80.6 (76.6, 84.6)		133.7 (129.9, 137.6)		78.1 (75.4, 80.8)		4.0 (3.7, 4.3)		55.2 (52.7, 57.6)	
Change from baseline	42	13.7 (10.0, 17.4)	<0.001	9.1 (4.9, 13.3)	<0.001	6.1 (2.7, 9.4)	<0.001	−0.1 (−0.3, 0.1)	0.43	−4.2 (−6.6, −1.8)	<0.001
% change from baseline	42	21.7 (16.2, 27.3)		7.8 (4.3, 11.3)		10.0 (4.8, 15.1)		−2.1 (−7.9, 3.7)		−6.8 (−10.8, −2.8)	
180 days	32	75.8 (72.0, 79.5)		135.1 (130.8, 139.4)		79.9 (76.5, 83.2)		4.2 (3.8, 4.5)		55.7 (53.3, 58.1)	
Change from baseline	32	11.9 (8.0, 15.8)	<0.001	10.8 (6.0, 15.5)	<0.001	8.5 (4.1, 12.8)	<0.001	0.2 (−0.1, 0.4)	0.18	−4.5 (−7.1, −1.9)	0.001
% change from baseline	32	20.1 (13.4, 26.7)		9.1 (5.1, 13.1)		13.4 (6.7, 20.2)		4.4 (−1.4, 10.2)		−7.1 (−11.3, −2.8)	

	LVEDVi			LVMi			LAVi		RVEF		RVEDVi	
	*n*	Mean (95% CI)	*P*	*n*	Mean (95% CI)	*P*	Mean (95% CI)	*P*	Mean (95% CI)	*P*	Mean (95% CI)	*P*
Baseline	49	79.0 (74.7, 83.3)		47	67.8 (63.2, 72.3)		41.0 (38.2, 43.7)		58.6 (56.9, 60.4)		76.8 (71.8, 81.8)	
28 days												
Change from baseline												
% change from baseline												
56 days												
Change from baseline												
% change from baseline												
84 days												
Change from baseline												
% change from baseline												
112 days	42	81.4 (76.9, 85.9)		40	72.7 (67.9, 77.5)		40.0 (37.2, 42.7)		57.9 (55.3, 60.4)		72.1 (68.1, 76.1)	
Change from baseline	42	3.9 (1.1, 6.7)	0.008	40	5.1 (2.8, 7.3)	<0.001	−0.1 (−2.4, 2.2)	0.95	−0.6 (−3.2, 2.0)	0.64	−2.5 (−5.9, 0.9)	0.15
% change from baseline	42	6.2 (2.1, 10.3)		40	8.9 (5.1, 12.7)		2.0 (−4.7, 8.8)		−0.5 (−4.9, 3.9)		−1.0 (−6.2, 4.2)	
180 days	30	82.3 (77.0, 87.5)		29	73.4 (67.5, 79.3)		40.7 (37.0, 44.5)		57.7 (55.3, 60.0)		70.8 (66.0, 75.6)	
Change from baseline	30	4.8 (0.1, 9.5)	0.05	29	4.1 (1.1, 7.2)	0.01	−0.3 (−3.4, 2.8)	0.84	0.9 (−1.3, 3.1)	0.41	−5.8 (−9.9, −1.6)	0.009
% change from baseline	30	8.1 (1.1, 15.1)		29	7.6 (3.0, 12.2)		1.3 (−6.3, 8.8)		2.0 (−1.9, 5.8)		−4.9 (−12.0, 2.1)	

CI, confidence interval; DBP, diastolic blood pressure; LAVi, left atrial volume indexed to body surface area; LVEDVi, left ventricular end‐diastolic volume indexed to body surface area; LVEF, left ventricular ejection fraction; LVMi, left ventricular mass indexed to body surface area; NT‐proBNP, N‐terminal pro‐brain natriuretic peptide; RVEDVi, right ventricular end‐diastolic volume indexed to body surface area; RVEF, right ventricular ejection fraction; SBP, systolic blood pressure.

### N‐terminal pro‐brain natriuretic peptide changes during withdrawal of specific therapies

**Figure 3:**
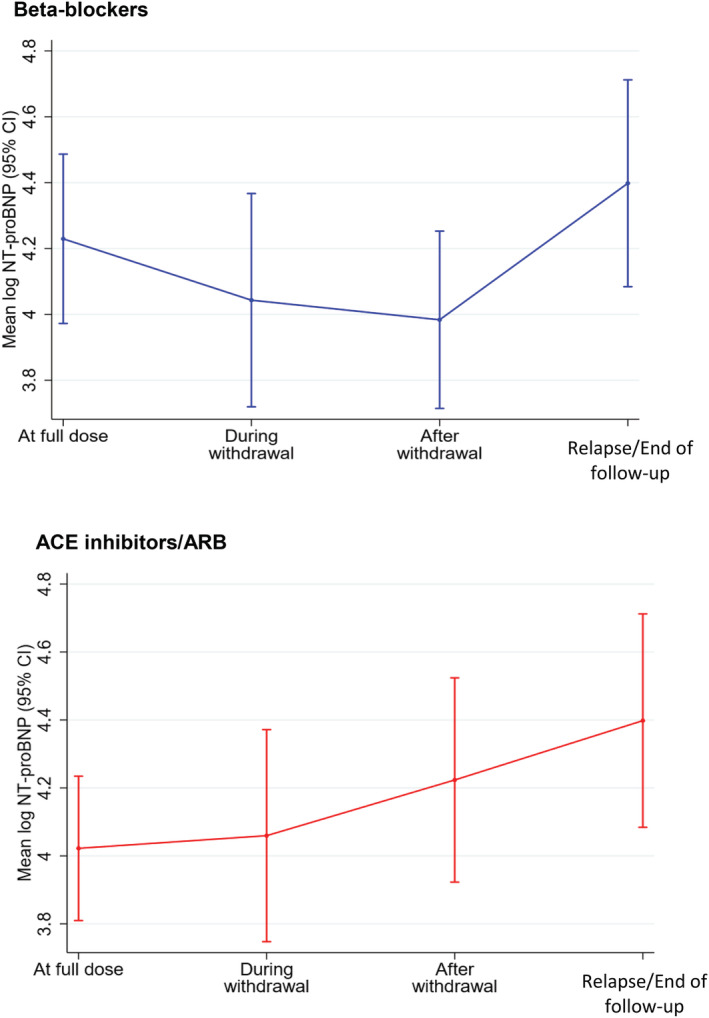
Mean log NT‐proBNP before, during, and immediately after withdrawal of beta‐blockers and ACE inhibitors or ARBs. ACE, angiotensin‐converting enzyme; ARB, angiotensin receptor blocker; CI, confidence interval; NT‐proBNP, N‐terminal pro‐brain natriuretic peptide.

There was a reduction in mean log NT‐proBNP during withdrawal of beta‐blocker therapy [change in log NT‐proBNP during withdrawal of beta‐blocker: −0.25 (95% CI −0.39, −0.10)] (*Figure* *3* and *Table* *3*), followed by a rise thereafter [change in log NT‐proBNP following withdrawal of beta‐blocker: 0.46 (95% CI 0.18, 0.73)]. As expected, the rise following withdrawal of beta‐blocker was greatest in those patients who relapsed compared with those who did not [0.85 (95% CI 0.39, 1.30) vs. 0.18 (95% CI −0.13, 0.50); *P* = 0.01].

**Table 3:** Change in log N‐terminal pro‐brain natriuretic peptide during and after withdrawal of specific medications

	Log NT‐proBNP						*P* ^a^
	All patients		Relapse		No relapse		
	*N*	Mean (95% CI)	*N*	Mean (95% CI)	*N*	Mean (95% CI)	
**Beta‐blockers**							
Before withdrawal	39	4.2 (4.0, 4.5)	16	4.5 (4.2, 4.9)	23	4.0 (3.7, 4.4)	
After withdrawal	39	4.0 (3.7, 4.3)	16	4.2 (3.7, 4.6)	23	3.9 (3.5, 4.2)	
Relapse/end of follow‐up	39	4.4 (4.1, 4.8)	16	5.0 (4.4, 5.7)	23	4.0 (3.7, 4.4)	
Change during withdrawal	39	−0.2 (−0.4, −0.1)	16	−0.4 (−0.6, −0.1)	23	−0.2 (−0.4, −0.01)	0.19
Change following withdrawal	39	0.5 (0.2, 0.7)	16	0.9 (0.4, 1.3)	23	0.2 (−0.1. 0.5)	0.01
**ACE inhibitor/ARB**							
Before withdrawal	46	4.0 (3.8, 4.2)	17	4.3 (3.8, 4.7)	29	3.9 (3.7, 4.1)	
After withdrawal	46	4.2 (3.9, 4.5)	17	4.8 (4.3, 5.4)	29	3.9 (3.6, 4.2)	
Relapse/end of follow‐up	46	4.4 (4.1, 4.7)	17	5.0 (4.4, 5.7)	29	4.0 (3.7, 4.3)	
Change during withdrawal	46	0.2 (−0.01, 0.4)	17	0.6 (0.2, 1.0)	29	−0.02 (−0.2, 0.2)	0.005
Change following withdrawal	46	0.2 (−0.06, 0.4)	17	0.2 (−0.2, 0.6)	29	0.2 (−0.2, 0.5)	0.82

ACE, angiotensin‐converting enzyme; ARB, angiotensin receptor blocker; CI, confidence interval; NT‐proBNP, N‐terminal pro‐brain natriuretic peptide.

^a^ *P*‐value from two‐sample *t*‐test comparing change in log NT‐proBNP between patients who relapsed and those who did not.

There were trends towards increases in mean log NT‐proBNP during and after withdrawal of ACE inhibitors or ARBs, although this did not reach statistical significance [change in log NT‐proBNP: during withdrawal of ACE inhibitor/ARB, 0.20 (95% CI −0.01, −0.41); following withdrawal of ACE inhibitor/ARB, 0.17 (95% CI −0.06, −0.41)]. The change in log NT‐proBNP following withdrawal of ACE inhibitor/ARB was not different between those who relapsed and those who did not [0.21 (95% CI −0.20, 0.62) vs. 0.15 (95% CI −0.15, 0.46); *P* = 0.82].

## Discussion

The TRED‐HF trial provides a unique opportunity to evaluate patients with DCM remission undergoing therapy withdrawal, prospectively and serially, using a standardized protocol with gold‐standard imaging investigations including CMR.^3^ The results provide a novel insight into the sequence of events during withdrawal of therapy and early adverse remodelling. This has important implications for how patients with DCM remission are monitored, supporting the use of simple clinical variables and imaging investigations to detect structural changes that occur early in the remodelling cascade. A rise in NT‐proBNP, a biomarker of congestion, typically occurs later. Reliance on natriuretic peptides to detect relapse may therefore miss a proportion of patients with deteriorating LV systolic function and LV hypertrophy. Indeed, over a third of patients who relapsed had a reduction in NT‐proBNP at this point and the majority had a reduction in LVEF of >10% at this point.

A rise in heart rate and blood pressure within 4–8 weeks after the start of therapy withdrawal reflects withdrawal of neurohormonal blockade and increasing sympathetic stimulation. The resulting increase in heart rate and afterload places greater workload on a vulnerable myocardium and appears likely to be the initial driver for relapse. Deterioration in LV systolic dysfunction and LV hypertrophy closely follow. It is possible that systolic dysfunction is caused by impaired energetic function, with insufficient adenosine triphosphate delivery caused by mitochondrial dysfunction and driven by increasing sympathetic stimulation.^10^, ^11^ Alternatively, sarcomeres may be vulnerable to myocardial loading with around one in five patients carrying truncating variants in *TTN*.^12^ The rapid increase in LV mass is unexpected. Only a few patients developed overt hypertension that was promptly treated with alternative anti‐hypertensives. This suggests that there was rapid up‐regulation of hypertrophic pathways and is in‐keeping with the findings of our analysis based on CMR relaxometry.^13^

The demonstration that NT‐proBNP typically rises months after the development of LV systolic dysfunction and hypertrophy is important for clinical practice. Increases in NT‐proBNP were seldom observed until a drop in LVEF of >10%. This suggests that the left ventricle can compensate for changes in systolic function for a prolonged period before ventricular and atrial stretch occurs and intracardiac pressures begin to rise. The late increase in LAVi, which was only found in patients who had met criteria for relapse, is in‐keeping with this concept.

The fall in NT‐proBNP after withdrawal of beta‐blockers is interesting. This supports the concept that beta‐blockers chronically increase plasma concentrations of NT‐proBNP, due to delayed myocardial relaxation, negative inotropic effects, or slower heart rate with consequent increase in left atrial volume and wall stress. We stress, however, that this does not support the withdrawal of beta‐blockers in such patients. These medications are likely to play an important role in the maintenance of heart failure remission. It is possible that increased secretion of natriuretic peptides might even contribute to the therapeutic action of beta‐blockers. Moreover, following the withdrawal of beta‐blockers, we observed a secondary increase in NT‐proBNP that was greatest amongst patients who relapsed. A similar observation was not seen following the withdrawal of ACE inhibitor/ARB. As heart failure therapy was not standardized at baseline and withdrawal of agents followed in quick succession, it is difficult to reliably determine the effects of withdrawing individual agents on different variables. We also recognize the limitations of these analyses with small numbers of patients and stress the exploratory nature of them.

The absence of clear changes in RVEF is in‐keeping with primary LV pathology. However, we have previously reported reduction in right ventricular (RV) global longitudinal strain amongst patients who had therapy withdrawal compared with those who continued therapy.^13^ Strain may be a more sensitive measure of early RV dysfunction to detect subtle changes in the RV. The reduction in RVEDVi seen at 6 months is interesting. Whether this is due to a change in LV geometry and increased LVEDVi is unclear.

The use of CMR to detect imaging changes provides gold‐standard reproducibility that affords the ability to detect subtle structural and functional changes in both the LV and RV within small numbers of patients. It is possible that more subtle changes in some measures, such as RVEF, may have been missed due to the relatively small sample size. Whilst it was not feasible to perform imaging at the same frequency as clinical examination and blood sampling, our protocol enabled us to demonstrate changes in mean LVMi and LVEF that clearly preceded change in NT‐proBNP. We have reported randomized comparisons of ventricular strain and tissue characterization markers; however, the current report is the first to demonstrate the time course of serial changes in LV and RV structure and function and how these relate to other clinical and biochemical variables.^13^ The same applies for the relationship between serial changes in heart rate and other clinical and imaging variables, which was not reported in previous work.^6^ This manuscript is therefore complementary and allows us to better understand the sequence of changes that occur during adverse remodelling and relapse. Clearly, variables used to define relapse cannot be used to predict relapse as that would be a self‐fulfilling prophecy. We have observed the sequence of events leading to changes in cardiac function that would lead most cardiologists to be concerned about the risk of relapse. This should help thoughtful physicians and patients to make better informed decisions about the management of DCM.

## Conclusions

We report for the first time that withdrawing therapy from asymptomatic patients with DCM and improved cardiac function leads to rapid increases in heart rate and blood pressure, which are accompanied or closely followed by changes in LV structure and function. Increases in NT‐proBNP typically occur later in the remodelling cascade, with increases in LAVi only seen amongst those with the most marked changes in ventricular structure or function. This confirms that tracking of simple clinical variables, such as heart rate, accompanied by regular imaging may be more effective than serial measurement of natriuretic peptides in detecting relapse.

## Conflict of interest

Prof. Pennell has received research support from Siemens and speaker's fees from Chiesi and Bayer and is a Director and owns stocks in Cardiovascular Imaging Solutions. Prof. Cowie reports consultancy work for AstraZeneca, Servier, Roche Diagnostics, and Amgen. Prof. Cleland reports personal fees from Abbott, grants and personal fees from Amgen, grants and personal fees from Bayer, personal fees and non‐financial support from Medtronic, grants and personal fees from Novartis, grants and personal fees from Pharmacosmos, grants and personal fees from Vifor, grants and personal fees from BMS, and grants and personal fees from Servier, outside the submitted work. All other authors have nothing to declare.

## Funding

The TRED‐HF was an investigator‐led trial sponsored by Royal Brompton and Harefield NHS Trust. The study was funded by a Clinical Research Training Fellowship from the British Heart Foundation (FS/15/29/31492) awarded to B.P.H. and S.K.P. and received additional support from the Alexander Jansons Foundation, the Cardiovascular Research Centre and NIHR Biomedical Research Unit at Royal Brompton Hospital, and the NIHR Imperial College Biomedical Research Centre and grants from Rosetrees Trust awarded to S.K.P. B.P.H. is also supported by a BHF Intermediate Fellowship (FS/ICRF/21/26019**)** and a Clinical Lecturer Starter Grant from the Academy of Medical Sciences (SGL021\1025). J.G.F.C. received support from a Centre of Research Excellence award from the British Heart Foundation (RE/18/6/34217).

## Supporting information




**Figure S1.** Mean change from baseline in variables stratified by the occurrence of relapse
**Table S1.** Summaries of variables over follow‐up stratified by relapse
