# Supplementary material for: Changes in clinical and imaging variables during withdrawal of heart failure therapy in recovered dilated cardiomyopathy
Source: ESC Heart Fail. 2022 Mar 8;9(3):1616–24. doi: 10.1002/ehf2.13872 (PMC9065828; doi:10.1002/ehf2.13872)
Supplement: Supplementary file 1 — Figure S1. Mean change from baseline in variables stratified by the occurrence of relapse Table S1. Summaries of variables over follow‐up stratified by relapse [file EHF2-9-1616-s001.docx]

**Serial changes in clinical, biochemical and imaging variables during withdrawal of heart failure therapy in patients with recovered dilated cardiomyopathy: an analysis from TRED-HF**

**Supplementary Figure 1. Mean change from baseline in variables stratified by the occurrence of relapse**


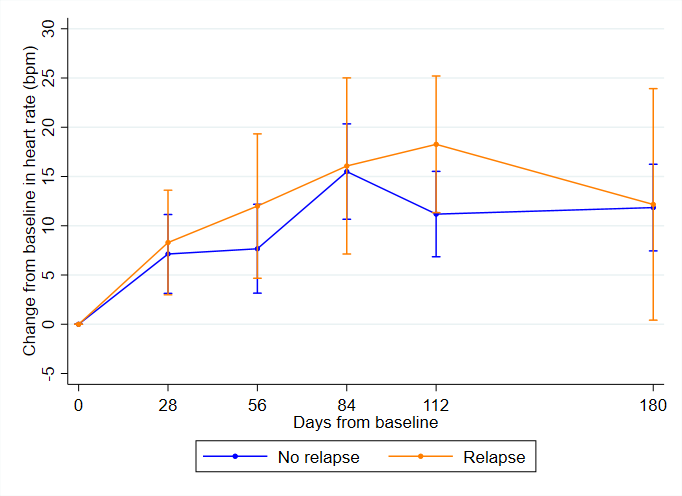

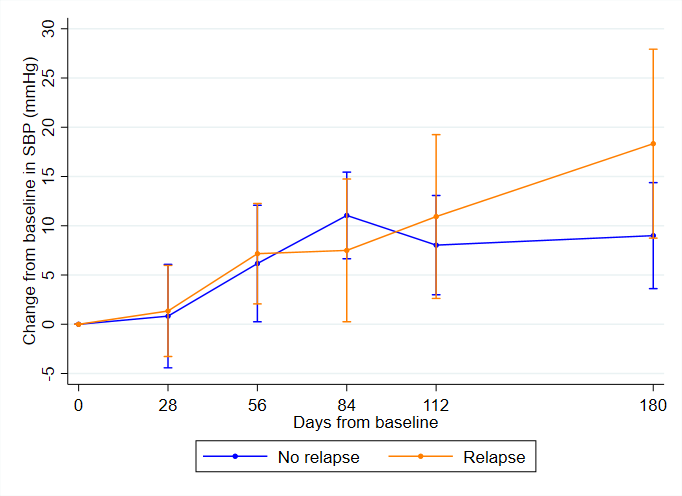


**Heart rate**

**Systolic BP**

**Diastolic BP**

**Log NT-pro-BNP**


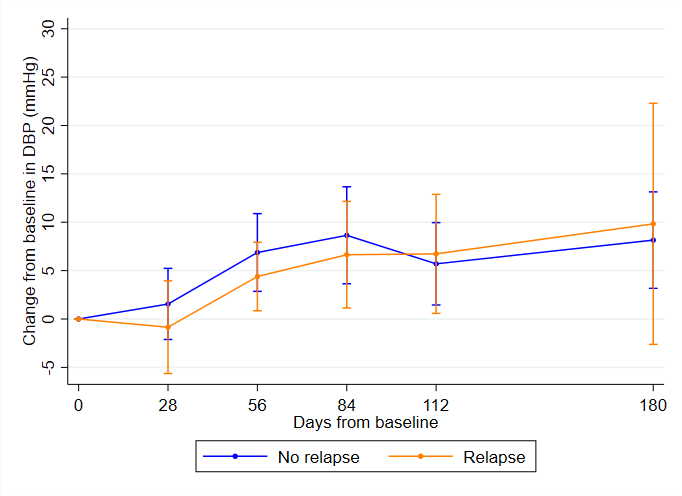

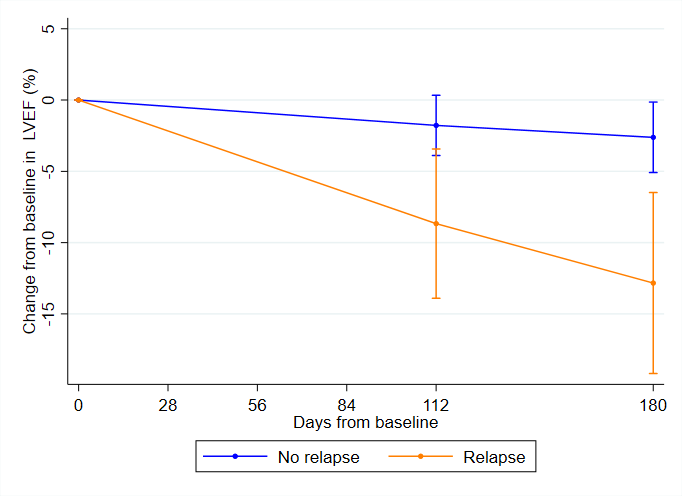

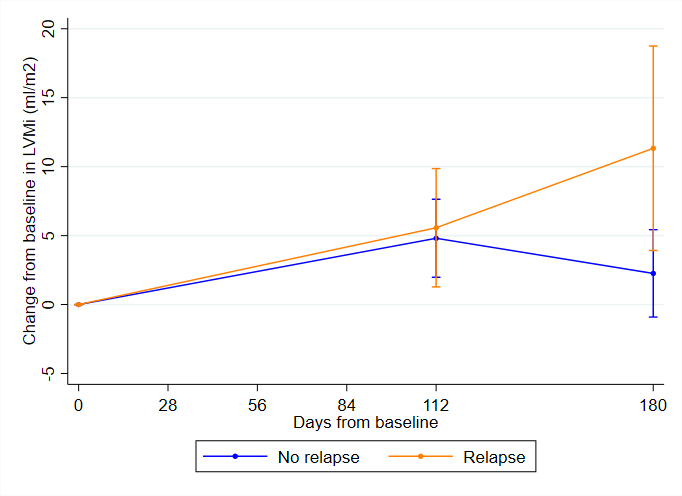

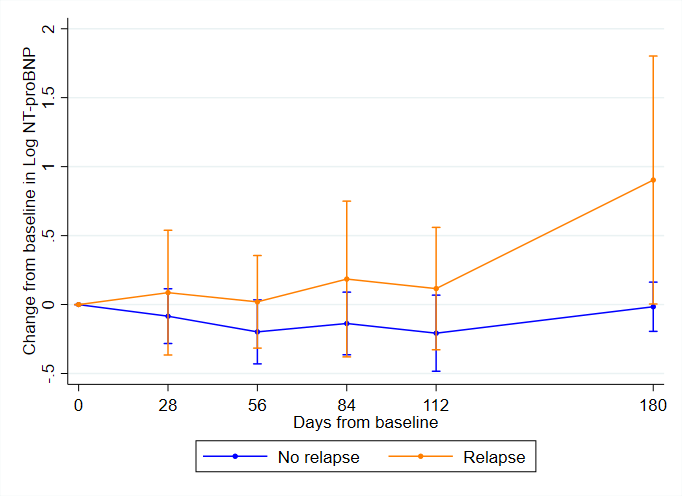


**LVEF**

**LV mass index**


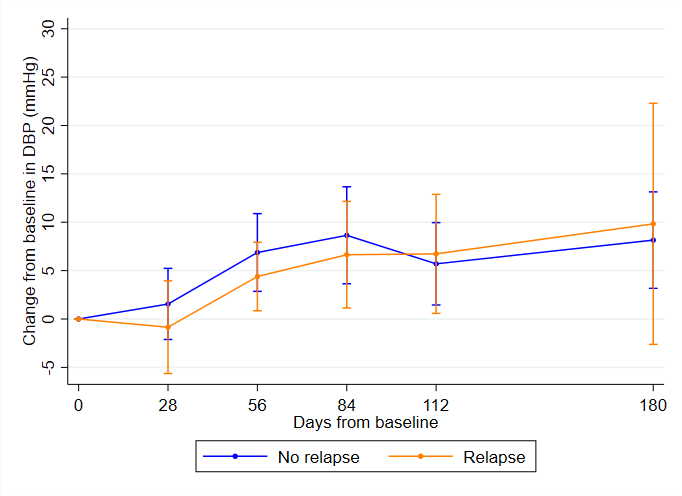

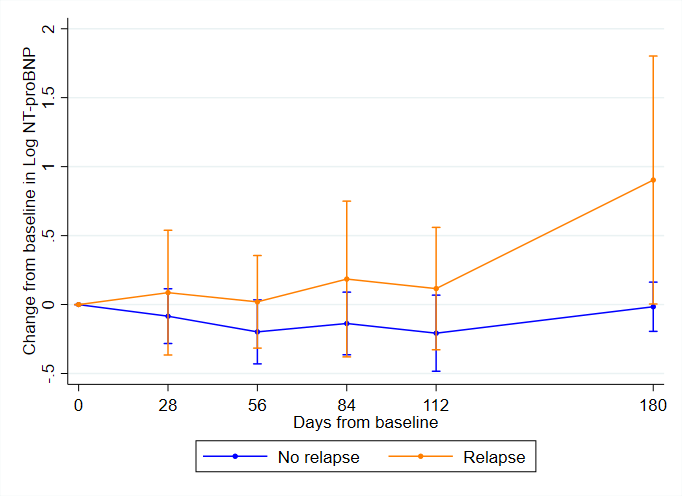

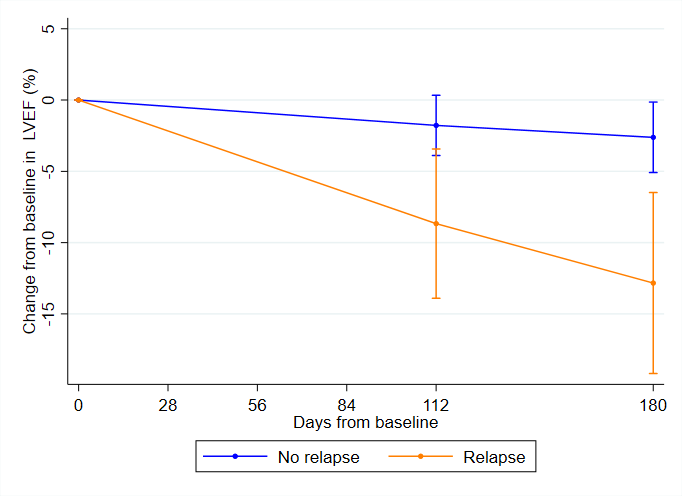

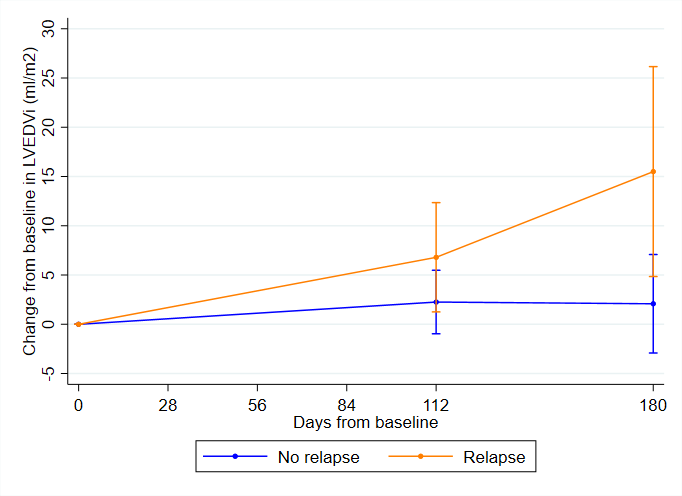

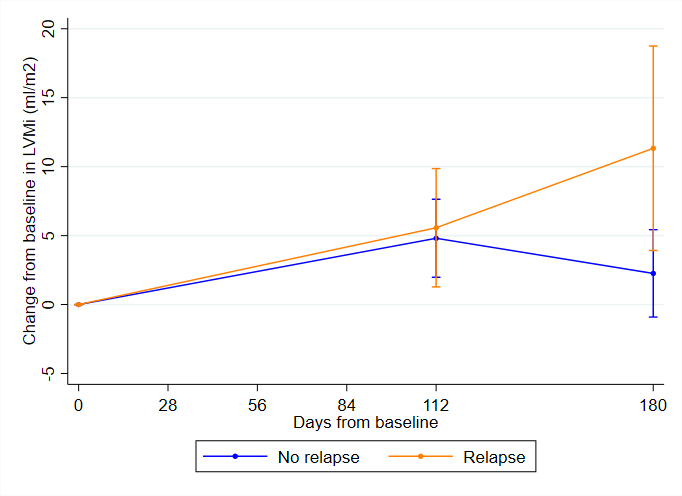

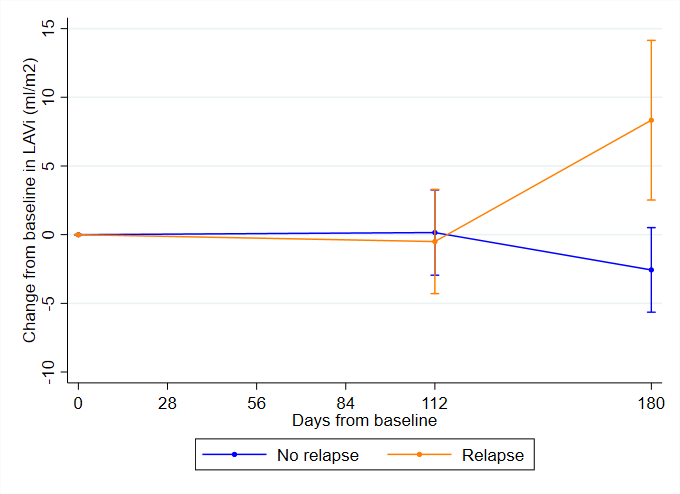

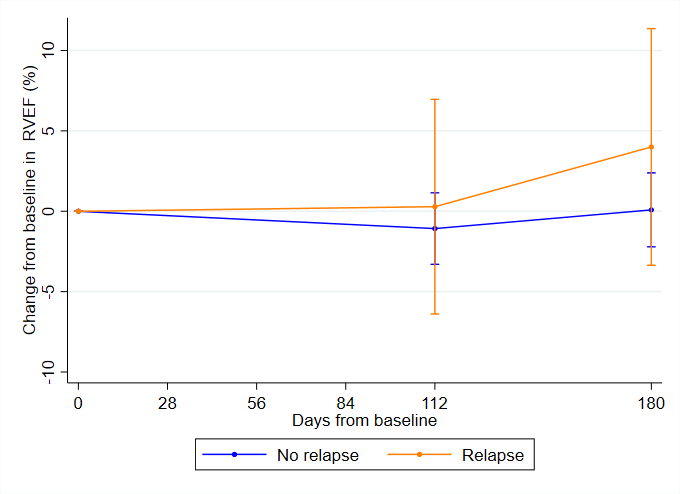

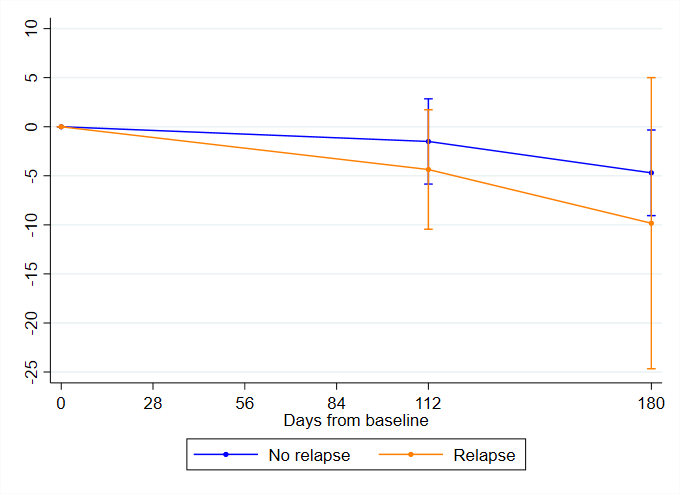


**Indexed LA volume**

**RVEF**

**Indexed LVEDV**


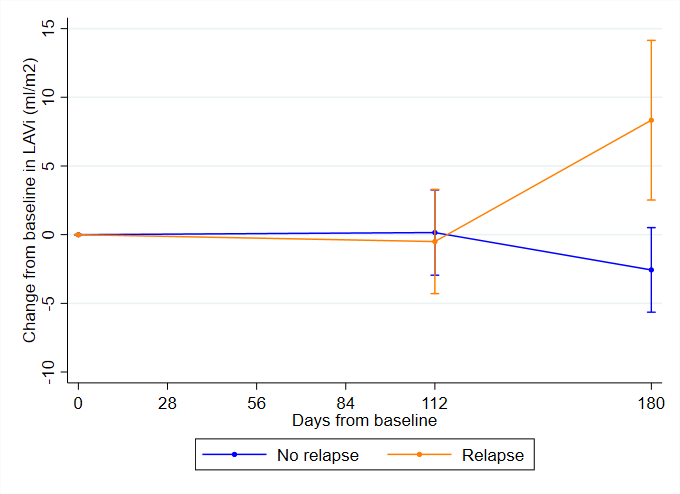

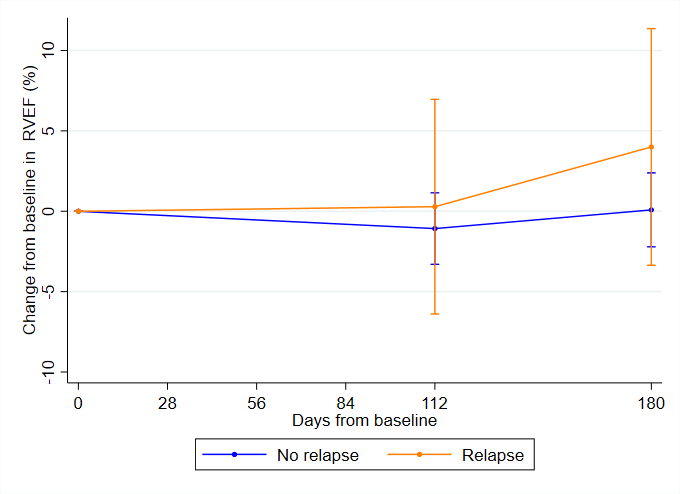

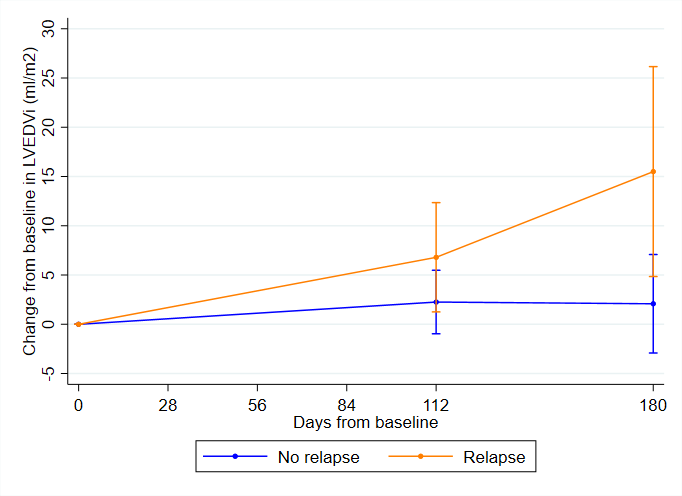

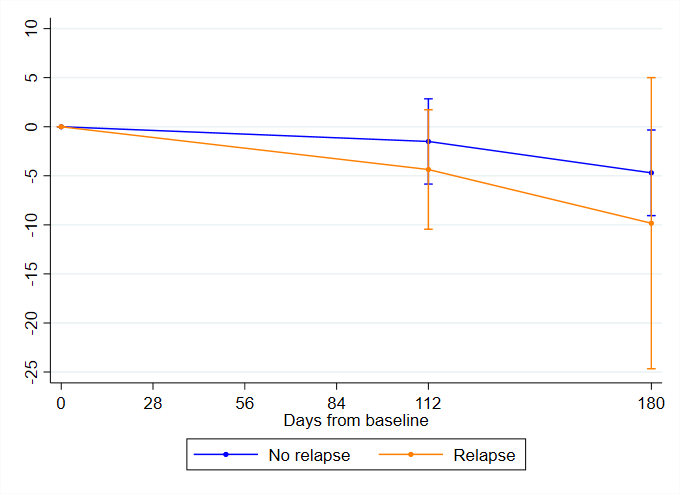


**Indexed RVEDV**


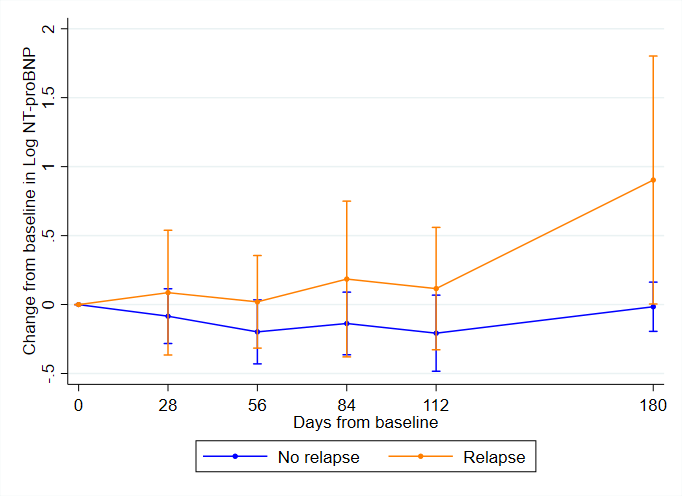

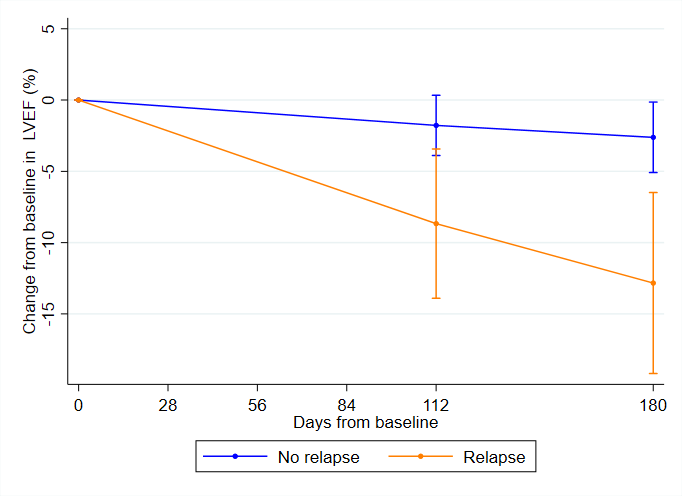

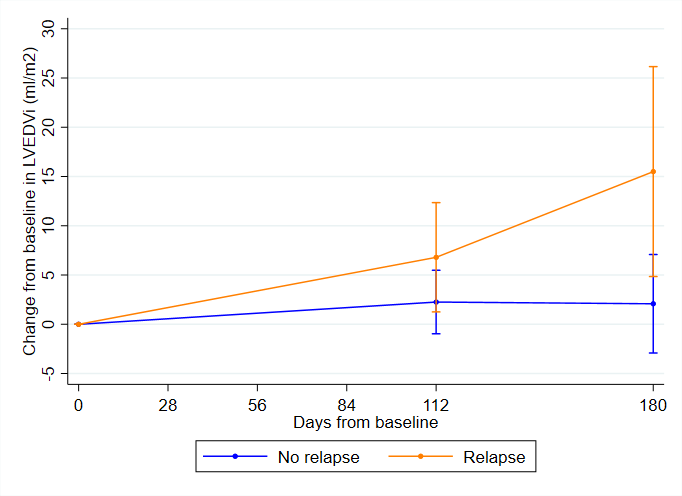

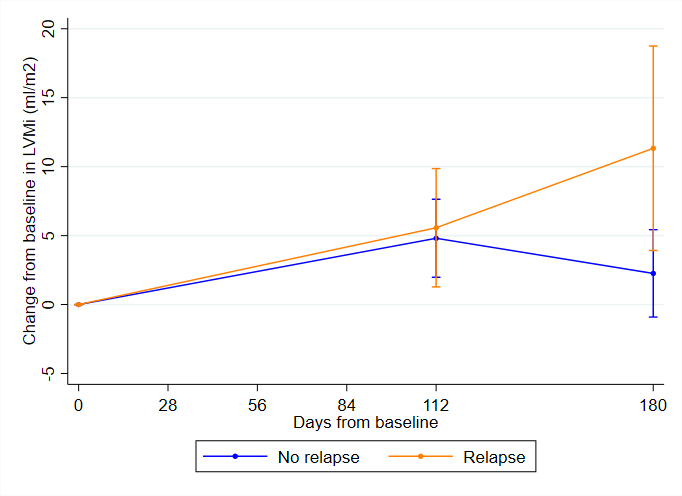

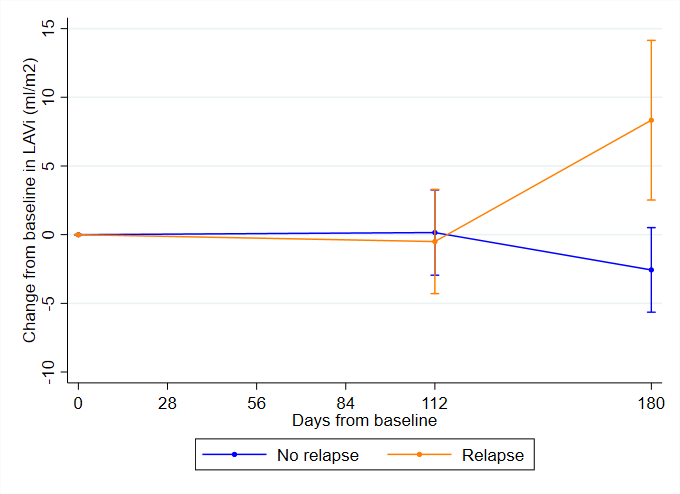

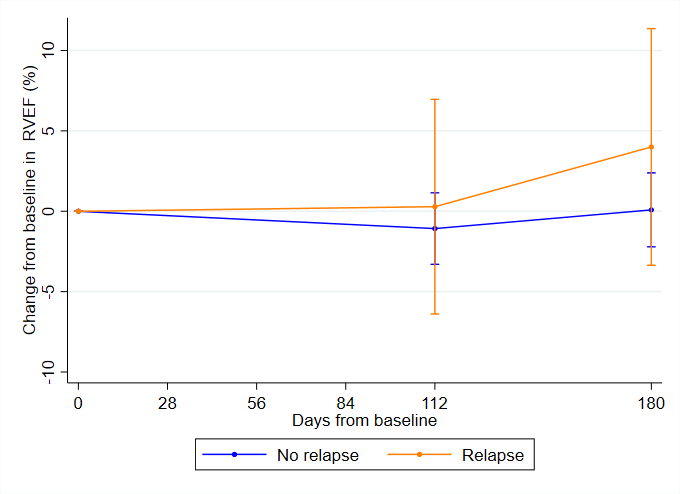

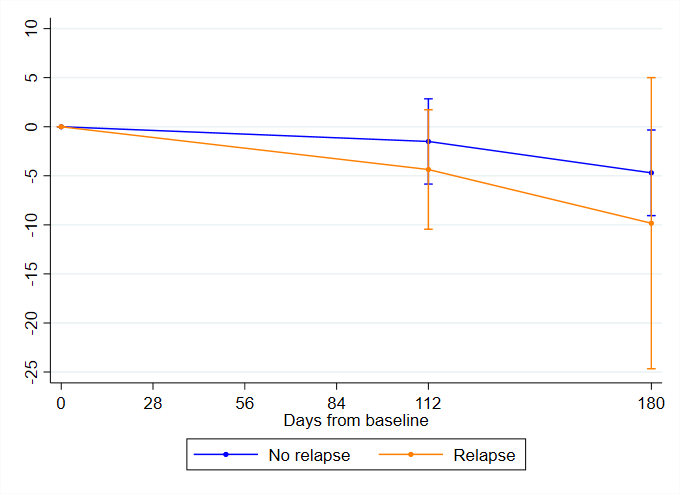


Values included until relapse or the end of the study

**Supplementary Table 1. Summaries of variables over follow-up stratified by relapse**

|  | **Heart rate** | | | | **Systolic BP** | | | | **Diastolic BP** | | | | **Log NT-pro-BNP** | | | | **LVEF** | | | |
| --- | --- | --- | --- | --- | --- | --- | --- | --- | --- | --- | --- | --- | --- | --- | --- | --- | --- | --- | --- | --- |
|  | **Relapse** | | **No relapse** | | **Relapse** | | **No relapse** | | **Relapse** | | **No relapse** | | **Relapse** | | **No relapse** | | **Relapse** | | **No relapse** | |
|  | **N** | **Mean (95% CI)** | **N** | **Mean (95% CI)** | **N** | **Mean (95% CI)** | **N** | **Mean (95% CI)** | **N** | **Mean (95% CI)** | **N** | **Mean (95% CI)** | **N** | **Mean (95% CI)** | **N** | **Mean (95% CI)** | **N** | **Mean (95% CI)** | **N** | **Mean (95% CI)** |
| **Baseline** | 20 | 68.3 (63.0, 73.6) | 29 | 64.6 (60.5, 68.7) | 20 | 122.9 (116.5, 129.3) | 29 | 124.8 (121.1, 128.4) | 20 | 71.5 (66.8, 76.1) | 29 | 72.9 (69.1, 76.6) | 20 | 4.5 (4.3, 4.8) | 29 | 4.0 (3.7, 4.3) | 20 | 59.0 (56.2, 61.9) | 29 | 60.5 (58.3, 62.8) |
| **28 days** | 20 | 76.6 (71.2, 82.0) | 29 | 71.8 (67.2, 76.3) | 20 | 124.3 (116.8, 131.7) | 29 | 125.6 (120.6, 130.5) | 20 | 70.6 (66.1, 75.1) | 29 | 74.4 (70.5, 78.4) | 20 | 4.6 (4.0, 5.2) | 29 | 3.9 (3.6, 4.2) |  |  |  |  |
| **Change from baseline** | 20 | 8.3 (3.0, 13.6) | 29 | 7.1 (3.1, 11.1) | 20 | 1.4 (-3.3, 6.0) | 29 | 0.8 (-4.4, 6.1) | 20 | -0.8 (-5.6, 3.9) | 29 | 1.6 (-2.1, 5.2) | 20 | 0.1 (-0.4, 0.5) | 29 | -0.1 (-0.3, 0.1) |  |  |  |  |
| **% change from baseline** | 20 | 13.8 (4.6, 23.0) | 29 | 12.0 (5.8, 18.2) | 20 | 1.2 (-2.6, 5.0) | 29 | 1.1 (-3.5, 5.7) | 20 | 0.0 (-7.6, 7.7) | 29 | 2.9 (-2.3, 8.2) | 20 | 1.2 (-8.8, 11.2) | 29 | -1.2 (-6.7, 4.3) |  |  |  |  |
| **56 days** | 18 | 81.8 (76.4, 87.2) | 24 | 73.3 (68.5, 78.1) | 18 | 131.7 (126.6, 136.7) | 24 | 130.1 (124.2, 136.1) | 18 | 75.7 (71.3, 80.1) | 24 | 79.2 (74.4, 83.9) | 18 | 4.5 (4.1, 4.9) | 24 | 3.9 (3.5, 4.2) |  |  |  |  |
| **Change from baseline** | 18 | 12.0 (4.7, 19.3) | 24 | 7.7 (3.2, 12.2) | 18 | 7.2 (2.1, 12.3) | 24 | 6.2 (0.3, 12.1) | 18 | 4.4 (0.8, 7.9) | 24 | 6.9 (2.9, 10.9) | 18 | 0.0 (-0.3, 0.4) | 24 | -0.2 (-0.4, 0.0) |  |  |  |  |
| **% change from baseline** | 18 | 19.8 (7.4, 32.2) | 24 | 12.9 (5.8, 20.0) | 18 | 6.3 (1.9, 10.6) | 24 | 5.3 (0.5, 10.2) | 18 | 6.8 (1.6, 12.1) | 24 | 10.2 (4.3, 16.1) | 18 | 0.4 (-7.3, 8.2) | 24 | -4.9 (-11.1, 1.2) |  |  |  |  |
| **84 days** | 14 | 84.3 (76.2, 92.4) | 20 | 80.4 (74.4, 86.4) | 14 | 133.1 (128.2, 138.0) | 20 | 134.6 (128.9, 140.2) | 14 | 79.3 (75.2, 83.4) | 20 | 81.3 (76.2, 86.4) | 14 | 4.7 (4.1, 5.3) | 19 | 4.0 (3.7, 4.4) |  |  |  |  |
| **Change from baseline** | 14 | 16.1 (7.1, 25.0) | 20 | 15.5 (10.7, 20.3) | 14 | 7.5 (0.3, 14.7) | 20 | 11.1 (6.7, 15.4) | 14 | 6.6 (1.1, 12.2) | 20 | 8.7 (3.6, 13.7) | 14 | 0.2 (-0.4, 0.7) | 19 | -0.1 (-0.4, 0.1) |  |  |  |  |
| **% change from baseline** | 14 | 25.9 (11.0, 40.8) | 20 | 24.8 (16.8, 32.9) | 14 | 6.7 (0.7, 12.7) | 20 | 9.1 (5.5, 12.8) | 14 | 10.3 (2.0, 18.7) | 20 | 13.0 (5.7, 20.3) | 14 | 5.0 (-7.6, 17.5) | 19 | -3.1 (-8.4, 2.2) |  |  |  |  |
| **112 days** | 15 | 88.5 (81.7, 95.2) | 27 | 76.2 (71.9, 80.5) | 15 | 135.5 (129.0, 142.0) | 27 | 132.8 (127.7, 137.9) | 15 | 77.5 (73.4, 81.7) | 27 | 78.5 (74.8, 82.2) | 15 | 4.5 (4.0, 5.1) | 27 | 3.7 (3.4, 4.0) | 15 | 49.8 (44.8, 54.8) | 27 | 58.1 (56.1, 60.2) |
| **Change from baseline** | 15 | 18.3 (11.3, 25.2) | 27 | 11.2 (6.9, 15.5) | 15 | 10.9 (2.6, 19.2) | 27 | 8.0 (3.0, 13.1) | 15 | 6.7 (0.6, 12.9) | 27 | 5.7 (1.4, 10.0) | 15 | 0.1 (-0.3, 0.6) | 27 | -0.2 (-0.5, 0.1) | 15 | -8.7 (-13.9, -3.4) | 27 | -1.8 (-3.9, 0.3) |
| **% change from baseline** | 15 | 27.2 (17.6, 36.9) | 27 | 18.7 (11.8, 25.6) | 15 | 9.6 (2.9, 16.4) | 27 | 6.8 (2.6, 11.0) | 15 | 11.4 (1.4, 21.4) | 27 | 9.2 (2.8, 15.5) | 15 | 2.6 (-7.6, 12.8) | 27 | -4.7 (-12.1, 2.6) | 15 | -14.4 (-22.9, -5.8) | 27 | -2.5 (-6.1, 1.0) |
| **180 days** | 6 | 79.7 (70.7, 88.6) | 26 | 74.8 (70.5, 79.1) | 6 | 140.8 (129.7, 151.9) | 26 | 133.8 (128.9, 138.6) | 6 | 78.5 (70.4, 86.6) | 26 | 80.2 (76.2, 84.1) | 6 | 5.1 (4.3, 6.0) | 26 | 3.9 (3.6, 4.2) | 6 | 48.0 (42.9, 53.1) | 26 | 57.5 (55.1, 59.8) |
| **Change from baseline** | 6 | 12.2 (0.4, 23.9) | 26 | 11.8 (7.4, 16.2) | 6 | 18.3 (8.7, 27.9) | 26 | 9.0 (3.6, 14.4) | 6 | 9.8 (-2.6, 22.3) | 26 | 8.2 (3.2, 13.1) | 6 | 0.9 (0.0, 1.8) | 26 | -0.0 (-0.2, 0.2) | 6 | -12.8 (-19.2, -6.5) | 26 | -2.6 (-5.1, -0.1) |
| **% change from baseline** | 6 | 19.5 (-0.5, 39.5) | 26 | 20.2 (12.7, 27.7) | 6 | 15.0 (7.0, 23.0) | 26 | 7.7 (3.1, 12.3) | 6 | 15.6 (-4.5, 35.7) | 26 | 13.0 (5.3, 20.6) | 6 | 22.5 (-0.8, 45.9) | 26 | 0.2 (-4.4, 4.8) | 6 | -20.7 (-29.0, -12.5) | 26 | -3.9 (-8.1, 0.2) |
|  |  |  |  |  |  |  |  |  |  |  |  |  |  |  |  |  |  |  |  |  |
|  | **LVEDVi** | | | | **LVMi** | | | | **LAVi** | | | | **RVEF** | | | | **RVEDVi** | | | |
|  | **Relapse** | | **No relapse** | | **Relapse** | | **No relapse** | | **Relapse** | | **No relapse** | | **Relapse** | | **No relapse** | | **Relapse** | | **No relapse** | |
|  | **N** | **Mean (95% CI)** | **N** | **Mean (95% CI)** | **N** | **Mean (95% CI)** | **N** | **Mean (95% CI)** | **N** | **Mean (95% CI)** | **N** | **Mean (95% CI)** | **N** | **Mean (95% CI)** | **N** | **Mean (95% CI)** | **N** | **Mean (95% CI)** | **N** | **Mean (95% CI)** |
| **Baseline** | 20 | 80.5 (74.3, 86.7) | 29 | 78.0 (71.9, 84.2) | 19 | 64.5 (57.0, 72.0) | 28 | 70.0 (64.1, 75.9) | 19 | 41.2 (37.5, 45.0) | 28 | 40.8 (36.8, 44.9) | 19 | 58.6 (55.9, 61.2) | 28 | 58.7 (56.2, 61.1) | 19 | 76.1 (68.8, 83.3) | 28 | 77.3 (70.1, 84.4) |
| **28 days** |  |  |  |  |  |  |  |  |  |  |  |  |  |  |  |  |  |  |  |  |
| **Change from baseline** |  |  |  |  |  |  |  |  |  |  |  |  |  |  |  |  |  |  |  |  |
| **% change from baseline** |  |  |  |  |  |  |  |  |  |  |  |  |  |  |  |  |  |  |  |  |
| **56 days** |  |  |  |  |  |  |  |  |  |  |  |  |  |  |  |  |  |  |  |  |
| **Change from baseline** |  |  |  |  |  |  |  |  |  |  |  |  |  |  |  |  |  |  |  |  |
| **% change from baseline** |  |  |  |  |  |  |  |  |  |  |  |  |  |  |  |  |  |  |  |  |
| **84 days** |  |  |  |  |  |  |  |  |  |  |  |  |  |  |  |  |  |  |  |  |
| **Change from baseline** |  |  |  |  |  |  |  |  |  |  |  |  |  |  |  |  |  |  |  |  |
| **% change from baseline** |  |  |  |  |  |  |  |  |  |  |  |  |  |  |  |  |  |  |  |  |
| **112 days** | 15 | 86.5 (76.6, 96.4) | 27 | 78.6 (74.1, 83.0) | 14 | 69.4 (59.3, 79.4) | 26 | 74.5 (69.1, 79.9) | 14 | 40.4 (35.7, 45.0) | 26 | 39.7 (36.0, 43.5) | 14 | 59.0 (53.0, 65.0) | 26 | 57.2 (54.6, 59.8) | 14 | 67.6 (59.2, 75.9) | 26 | 74.5 (70.1, 79.0) |
| **Change from baseline** | 15 | 6.8 (1.3, 12.3) | 27 | 2.3 (-1.0, 5.5) | 14 | 5.6 (1.3, 9.9) | 26 | 4.8 (2.0, 7.6) | 14 | -0.5 (-4.3, 3.3) | 26 | 0.2 (-3.0, 3.3) | 14 | 0.3 (-6.4, 7.0) | 26 | -1.1 (-3.3, 1.1) | 14 | -4.4 (-10.4, 1.7) | 26 | -1.5 (-5.8, 2.8) |
| **% change from baseline** | 15 | 8.7 (0.8, 16.7) | 27 | 4.8 (-0.2, 9.8) | 14 | 10.0 (1.8, 18.3) | 26 | 8.3 (4.1, 12.5) | 14 | 0.9 (-10.3, 12.1) | 26 | 2.6 (-6.3, 11.6) | 14 | 1.3 (-10.1, 12.7) | 26 | -1.5 (-5.3, 2.3) | 14 | -5.1 (-13.4, 3.2) | 26 | 1.2 (-5.8, 8.1) |
| **180 days** | 6 | 91.5 (82.4, 100.6) | 24 | 80.0 (73.9, 86.0) | 6 | 73.2 (47.4, 98.9) | 23 | 73.4 (67.8, 79.1) | 6 | 52.0 (40.7, 63.3) | 23 | 37.8 (34.6, 40.9) | 6 | 60.0 (50.5, 69.5) | 23 | 57.1 (54.8, 59.4) | 6 | 59.8 (53.3, 66.4) | 23 | 73.7 (68.4, 79.0) |
| **Change from baseline** | 6 | 15.5 (4.8, 26.2) | 24 | 2.1 (-2.9, 7.1) | 6 | 11.3 (3.9, 18.7) | 23 | 2.3 (-0.9, 5.4) | 6 | 8.3 (2.5, 14.1) | 23 | -2.6 (-5.6, 0.5) | 6 | 4.0 (-3.4, 11.4) | 23 | 0.1 (-2.2, 2.4) | 6 | -9.8 (-24.7, 5.0) | 23 | -4.7 (-9.1, -0.3) |
| **% change from baseline** | 6 | 21.7 (4.1, 39.4) | 24 | 4.6 (-2.9, 12.2) | 6 | 19.1 (6.6, 31.6) | 23 | 4.6 (0.1, 9.1) | 6 | 18.8 (7.1, 30.4) | 23 | -3.3 (-11.6, 4.9) | 6 | 7.3 (-4.9, 19.5) | 23 | 0.6 (-3.5, 4.7) | 6 | -11.4 (-30.3, 7.5) | 23 | -3.2 (-11.4, 4.9) |

Relapse: n=20; non-relapse: n=29. DBP: diastolic blood pressure; LAVi: left atrial volume indexed to body surface area; LVEF: left ventricular ejection fraction; LVEDVi: left ventricular end-diastolic volume indexed to body surface area; LVMi: left ventricular mass indexed to body surface area; RVEDVi – right ventricular end-diastolic volume indexed to body surface area; RVEF – right ventricular ejection fraction; SBP – systolic blood pressure;
